# Eggs

**Published:** 1890-05

**Authors:** 


### EGGS.

The egg is a most concentrated form of food, and very nearly a perfect food. Never limit the use of eggs, for though they be high priced, they are cheaper than meat and very nutritious. This does not mean that they should be eaten exclusively or used extravagantly in making rich cakes and custards. They should be served in the numberless ways in which eggs as a food have found no equal. It is very poor economy to use half-cooked flour in an omelet, or soda and cream of tartar in sponge cake, as a substitute for eggs.

The white of the egg, or the albumen, consists of 85 parts water, 12 parts pure albumen, 2.7 parts mucus, and of saline matter 0.3, including soda. The white of egg has scarcely any fat, and in using requires butter, milk or fat meat.

Why does the white of the egg increase in bulk when beaten? The pure albumen is enclosed in cells which break when beaten. Albumen is a glutinous substance; this catches and holds the air and increases its bulk many times. To housekeepers fortunate enough to obtain their eggs direct from the farmer or those having hens of their own, I would caution the use of an egg before it has been laid ten hours; the white has not become set and thick and cannot be beaten stiff. The white of the egg is almost indispensable in clarifying soups jellies, and coffee, since the albumen, affected by the heat, hardens and draws within itself any solids or impurities and either rises to the surface or sinks to the bottom with them, leaving the liquid pure and clear.

The yolk of the egg contains the same constituents as the white with the addition of oil and sulphur.

The longer eggs are kept the lighter they become; therefore, if in doubt about the worth of an egg, try it in a cup of water. If the egg rises to the surface and swims, it is not good, and should not be used. The shell of a fresh egg is nearly full, but the shell is porous and the water in the egg evaporates, while fresh air fills its place, and the egg soon spoils.

There are many excellent and quite reliable ways of preserving eggs. Any substance that will fill the pores in the shell and exclude air, will preserve eggs indefinitely. Eggs for preserving must be perfectly fresh. Many persons preserve eggs by packing in bran, salt, etc., with the small end of the egg down. This may keep them sound and good if attended with great care; and still I think one can be less sure in this method if one desires to preserve the eggs for any great length of time. My own experience has been that a coating of varnish thoroughly applied, will keep eggs for any length of time. After varnishing and drying, pack in a box of clean sawdust, and keep them where it is cool. The varnish has many substitutes, as lard, gum, wax, etc., but white varnish is the more satisfactory, as the eggs are always pleasant to handle, and the shell will break very evenly. A very good Scotch method is to drop the eggs for two minutes in boiling water. The heat coagulates the membrane within the shell, and renders it impervious to air.—*Good Housekeeping*.

